# Ecotoxicological Effects of a Biomass-Derived Carbon Adsorbent on the Mussel *Mytilus galloprovincialis*

**DOI:** 10.3390/ijms27146358

**Published:** 2026-07-17

**Authors:** Ângela Almeida, Tiago Canha, Marta Cunha, Vânia Calisto, Rosa Freitas

**Affiliations:** 1 Department of Chemistry and Centro de Estudos do Ambiente e do Mar (CESAM), University of Aveiro, 3810-193 Aveiro, Portugal; vania.calisto@ua.pt; 2 Department of Physics, University of Aveiro, 3810-193 Aveiro, Portugal; tiagocanha1@ua.pt; 3 Department of Biology and Centro de Estudos do Ambiente e do Mar (CESAM), University of Aveiro, 3810-193 Aveiro, Portugal; martacunha@ua.pt (M.C.); rosafreitas@ua.pt (R.F.)

**Keywords:** carbon-based materials, biomarkers, ecotoxicity, invertebrates, sub-lethal effects

## Abstract

Carbon-based materials like activated carbon (AC) are frequently applied for water treatments and remediation. The increasing use and functionalization of AC, especially with the recent mandate to implement quaternary treatments to remove organic micropollutants (Directive 2024/3019), may inadvertently introduce AC or leachate products to aquatic bodies. Such occurrences pose potential risks to inhabiting organisms, which have been understudied. This study assessed the environmental safety of an AC obtained from spent brewery grains (SBG)—a lignocellulosic biomass—through microwave pyrolysis with potassium carbonate activation. The resulting AC (SBG-AC) was washed, sieved (powder, particle size ≤ 180 µm), and tested for its ecotoxicological effects on the marine mussel *Mytilus galloprovincialis* at doses of 5, 25, and 50 mg/L. After 28 days of exposure (with weekly water renewal), biochemical parameters related to the mussels’ metabolic capacity and oxidative status were evaluated. Exposure to SBG-AC stimulated the energy metabolism in *M. galloprovincialis*, at the expense of internal energy reserves (such as glycogen). Although SBG-AC exposure induced antioxidant responses, the significant increase in lipid peroxidation and protein carbonylation at the higher doses (particularly 50 mg/L) suggests that these protective mechanisms were insufficient to prevent oxidative damage. Overall, while SBG-AC offers an effective alternative for water treatment, its ecotoxicity at higher doses raises concerns, emphasizing the need for careful risk assessment and containment measures.

## 1. Introduction

Carbon-based materials, such as activated carbon (AC) and biochar, are widely used across various fields due to their low cost, high surface area, porous structure, and versatility. These materials have been applied to enhance soil quality and for removal of contaminants from air and water, as support for developing catalysts, and in carbon sequestration and manufacture of other carbon-based materials such as biocomposites, electrochemical devices, biomedical products, and cosmeceuticals (reviewed by [1,2]). Despite their broad utility, these materials still face challenges in terms of sustainable production and recovery/reuse (e.g., [3]). In addition, the increasing use and further functionalization of carbon-based materials, for example through the incorporation of acidic or basic groups or metal ions to improve material performance [4], may inadvertently introduce them or leachate products into aquatic bodies, potentially impacting inhabiting organisms. The environmental relevance of these concerns is particularly evident in the context of water treatment. Activated carbon and biochar are among the most widely applied adsorbents for removing organic contaminants, such as pharmaceuticals, dyes, metals, and other persistent compounds [5,6,7,8,9,10,11,12]. However, their large-scale use must be aligned with the recent directive (Directive EU 2024/3019) on urban wastewater treatment, concerning the implementation of quaternary treatments for organic compound removal [13]. This framework drives the search for improved carbon-based adsorption materials, but developing cost-effective and eco-friendly water treatment methodologies is as important as minimizing contaminants’ entrance/dissemination into aquatic systems. Consequently, it requires a comprehensive evaluation of the potential ecotoxicological impacts of the treatment agents themselves.

Studies have reported that the application of carbon-based materials may have adverse effects on aquatic organisms, with most of the works being focused on in situ impacts of soil amendments with AC [14,15,16,17,18]. Both positive and negative implications have been reported in the literature, raising the importance of assessing ecotoxicity testing before application. For example, studies reported adverse effects of AC on aquatic and sediment benthic invertebrates, including reduced survival, growth, and altered behavior; among the tested items were clams (*Macoma balthica*), oligochaetes/polychaetes (*Lumbriculus variegatus*, *Marenzelleria* spp.), and crustaceans (*Corophium volutator*, *Daphnia magna*) [16,19,20]. The responses were related to size-dependent ingestion of AC particles, adsorption of nutritious organic matter, and sediment property alterations such as porewater pH shifts that disrupt waterborne microbial and meiofaunal communities [21]. The literature reports a dual effect of AC, depending mostly on materials’ particle size and dose (generally up to 4% (*w*/*w*) of the sediment weight). In fact, Rämö et al. [16] observed that responses to commercial AC (anthracite-based) were species-specific and varied with particle size, as follows: powdered AC (PAC, ingestible) resulted in starvation through reduced bioavailability of food or rejection of AC-treated sediment; granular AC (GAC, non-ingestible) led to an increase in both dry weight and carbon assimilation. Still concerning the size of AC, Kupryianchyk et al. [22] concluded that in situ PAC should be used at sites with high contaminant concentrations and urgent ecosystem protection needs, whereas GAC is adequate for vulnerable ecosystems where longer-term protection may be sufficient.

Besides AC, biochar is another carbon-based material produced through the thermal degradation of carbon-rich precursors, including biomass [10,23]. Previous studies have reported that biochar induces oxidative stress, cell aggregation, neurotoxicity, and growth impairment in different aquatic organisms such as freshwater algae (*Scenedesmus obliquus*) [24], nematodes (*Caenorhabditis elegans*) [25], and other invertebrates (the ciliate *Paramecium caudatum*, the rotifer *Lecane quadridentata*, and the crustaceans *D. magna* and *Moina macrocopa*) [26]. The toxicity was related to the presence of persistent free radicals and hazardous compounds, such as polycyclic aromatic hydrocarbons (PAHs) and polychlorinated dibenzo-p-dioxins and furans, on the surfaces of the carbon-based materials, which were formed during the thermal degradation of biomass used for their production [12]. However, AC is generally produced under more severe conditions than biochar [10,27], which may reduce the presence of volatile and semi-volatile organic residues, including PAHs, in the final material. Nevertheless, depending on the production and post-treatment conditions, AC may retain oxygen-containing surface functional groups [28] which can participate in redox reactions and may contribute to oxidative stress.

To address knowledge gaps on the environmental impacts of AC via waterborne exposure (alternative pathways) in aquatic systems, the present study investigated the environmental safety of an AC obtained by microwave pyrolysis of spent brewery grains (SBG), a lignocellulosic waste. The AC (SBG-AC) produced (≤180 µm) was tested for its ecotoxicological effects on the marine mussel species *Mytilus galloprovincialis* at doses of 5, 25, and 50 mg/L. After 28 days of exposure to the referred doses (with weekly water renewal), were evaluated the following biochemical parameters: (i) the organism metabolic capacity (electron transport system activity, glycogen content, and total protein content); (ii) the oxidative status—enzymatic and non-enzymatic antioxidant defenses (superoxide dismutase, catalase, glutathione peroxidase, and total antioxidant capacity), (iii) oxidative damage (lipid peroxidation and protein carbonylation levels), and neurotoxicity (acetylcholinesterase activity). Based on the particulate nature of SBG-AC, it was hypothesized that SBG-AC exposure could affect mussel physiology by modifying filtration capacity, increasing metabolic demands, and altering redox homeostasis. Aquatic organisms rely on antioxidant defenses (scavengers and enzymes) to overcome the adverse effects exerted by reactive oxygen species (ROS), usually produced during several cellular pathways (e.g., the oxidative phosphorylation) [29]. However, environmental stressors may alter redox homeostasis by elevating ROS generation and/or modifying antioxidant responses, which can manifest as either compensatory upregulation or inhibition of antioxidant activity, ultimately causing lipid peroxidation (LPO), protein carbonylation (PC), and DNA damage.

The selected exposure period was chosen to represent a sub-chronic exposure scenario, allowing the detection of biochemical responses before the onset of severe toxicity. This duration is commonly applied in bivalve ecotoxicological studies, allowing particle interaction, ingestion, and biological responses [30,31,32]. The tested SBG-AC doses were selected based on previous wastewater treatment studies that used this material as an adsorbent for contaminant removal, particularly pharmaceutical drugs [8,33]. *M. galloprovincialis* mussels were selected as the test organisms due to their ecological relevance and established use as a sentinel species in marine pollution monitoring [34,35]. Its suspension-feeding behavior makes it suitable for assessing the effects of particulate materials such as SBG-AC, since mussels potentially accumulate particles from the water column and retain them in tissues [36]. Given the potential for carbon-based materials to enter aquatic environments and impact estuarine or marine ecosystems, further evaluation of their ecotoxicological effects is essential. To our knowledge, this is the first study investigating the toxicological impacts of an AC derived from SBG, a widely available lignocellulosic byproduct, and produced via microwave pyrolysis, an emerging energy-efficient technology. The findings provide a basis for selecting AC doses that minimize impacts on aquatic organisms, supporting their safe application within the framework of water policy.

## 2. Results

### 2.1. Energy-Related Parameters

**Figure 1:**
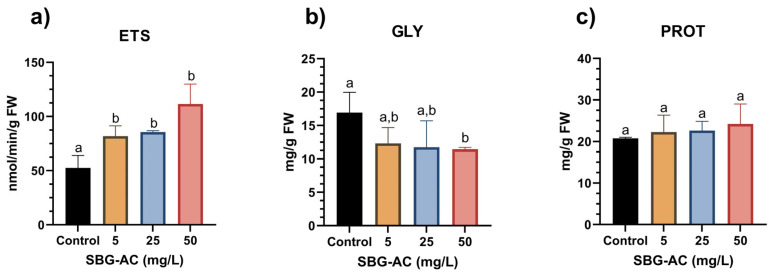
Energy-related parameters: (**a**) ETS, electron transport system activity; (**b**) GLY, glycogen content; (**c**) PROT, total protein content in *M. galloprovincialis* exposed to different doses of SBG-AC (control, 5, 25 and 50 mg/L) for 28 days. Results are the mean ± standard deviation, but for visual clarity, only the upper (+) standard deviation error bars are displayed. Bars with different lowercase letters indicate significant differences among treatments (*p* < 0.05), whereas bars with the same letter indicate no significant differences.

The results of energy-metabolism are depicted in Figure 1. Concerning electron transport system (ETS) activity (Figure 1a), a significant increase in metabolic activity in mussels exposed to SBG-AC (all doses) was observed compared to the control treatment, with a dose-dependent response. The results of glycogen (GLY) (Figure 1b) showed a general decrease in its content in SBG-AC-exposed mussels but only differed statistically when comparing the highest SBG-AC dose (50 mg/L) with the control. In terms of the total protein (PROT) content, no significant differences were observed in mussels exposed to SBG-AC at all doses when compared with the control, although SBG-AC-exposed organisms presented higher PROT content than CTL ones (Figure 1c).

### 2.2. Indicators of Antioxidant Responses

**Figure 2:**
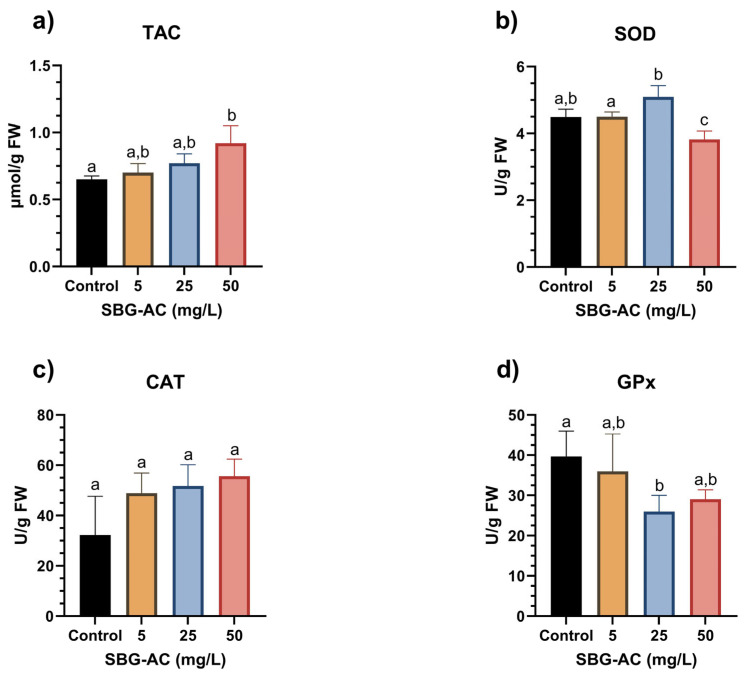
Antioxidant indicators: (**a**) TAC, total antioxidant capacity; (**b**) SOD, superoxide dismutase activity; (**c**) CAT, catalase activity; (**d**) GPx, glutathione peroxidase activity in *M. galloprovincialis* exposed to different doses of SBG-AC (control, 5, 25, and 50 mg/L) for 28 days. Results are the mean ± standard deviation, but for visual clarity, only the upper (+) standard deviation error bars are displayed. Bars with different lowercase letters indicate significant differences among treatments (*p* < 0.05), whereas bars with the same letter indicate no significant differences.

The results of antioxidant responses are depicted in Figure 2. Concerning total antioxidant capacity (TAC) (Figure 2a), the results showed that mussels exposed to SBG-AC overall increased the total antioxidant capacity but only differed statistically comparing the highest SBG-AC dose (50 mg/L) with the control. The activity of superoxide dismutase (SOD) (Figure 2b) showed a significant increase at SBG-AC 25 mg/L compared to 5 mg/L; however, it did not differ from the control condition. At the highest exposure dose (50 mg/L), SOD activity decreased significantly compared to the remaining treatments. The results of the catalase (CAT) assay (Figure 2c) showed that enzyme activity increased in the presence of SBG-AC, although no statistically significant difference was observed compared with the control. The activity of glutathione peroxidase (GPx) (Figure 2d) revealed a general decrease in mussels exposed to higher SBG-AC doses (25 and 50 mg/L) but only differed significantly when comparing the 25 mg/L and control treatments.

### 2.3. Indicators of Cellular Damage

**Figure 3:**
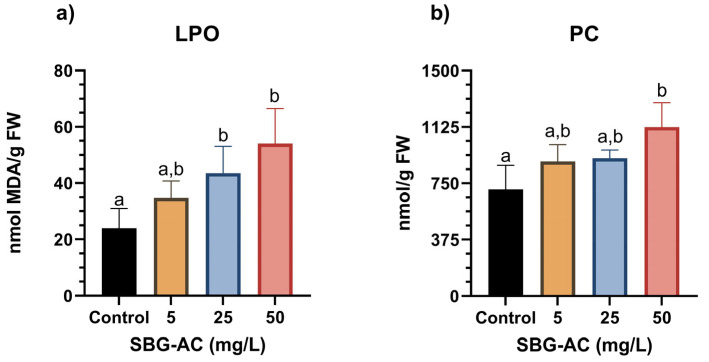
Indicators of cellular damage: (**a**) LPO, lipid peroxidation levels; (**b**) PC, protein carbonylation levels in *M. galloprovincialis* exposed to different doses of SBG-AC (control, 5, 25, and 50 mg/L) for 28 days. Results are the mean ± standard deviation, but for visual clarity, only the upper (+) standard deviation error bars are displayed. Bars with different lowercase letters indicate significant differences among treatments (*p* < 0.05), whereas bars with the same letter indicate no significant differences.

Indicators of cellular damage are represented in Figure 3. Accordingly, lipid peroxidation (LPO) levels increased with SBG-AC dose (Figure 3a), with significantly higher values than the control at 25 and 50 mg/L treatments. Also, protein carbonylation (PC) levels (Figure 3b) increased in SBG-AC-exposed mussels, with significant differences between the highest dose of SBG-AC (50 mg/L) and the control.

### 2.4. Indicator of Neurotoxicity

**Figure 4:**
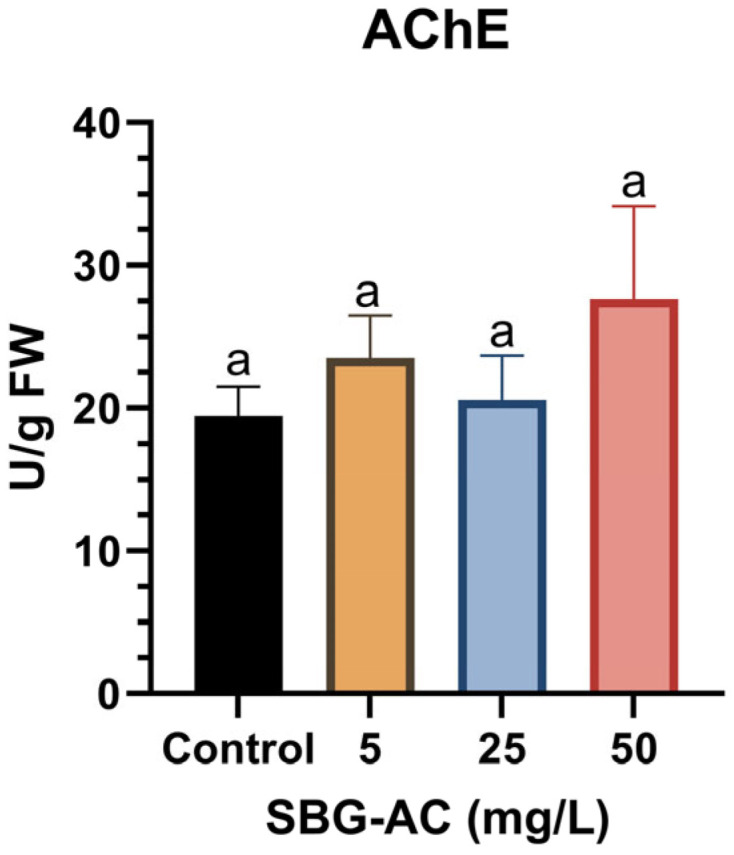
Indicators of neurotoxicity: AChE, acetylcholinesterase activity in *M. galloprovincialis* exposed to different doses of SBG-AC (control, 5, 25 and 50 mg/L) for 28 days. Results are the mean ± standard deviation, but for visual clarity, only the upper (+) standard deviation error bars are displayed. Bars with different lowercase letters indicate significant differences among treatments (*p* < 0.05), whereas bars with the same letter indicate no significant differences.

The response of a neurotoxicity indicator—acetylcholinesterase activity (AChE)—is shown in Figure 4. The results did not show significant differences among treatments; however, the activity of this enzyme appeared to increase in the presence of SBG-AC, particularly at the highest concentration (50 mg/L).

## 3. Discussion

Carbon-based materials such as activated carbon (AC) from spent brewery grains (SBG) (SBG-AC) are promising for diverse applications in water treatment/remediation strategies [37]; yet their potential ecotoxicological impacts remain insufficiently explored. In water treatment, AC has been applied for the removal of trace metals, pesticides, dyes, and pharmaceutical drugs [6,7,8,11], acting as a frontline barrier/remediation strategy against contaminants release and dissemination into aquatic systems. In particular, SBG-AC has been used to adsorb pharmaceutical compounds from water and wastewater, effectively removing antibiotics and antiepileptics at adsorbent doses between 15 and 50 mg/L (e.g., [8,9,10,23]). According to Directive (EU) 2024/3019, WWTPs with capacities of at least 150,000 population equivalents (p.e.) and agglomerations of at least 10,000 p.e. are required to apply a quaternary (advanced) treatment for the removal of organic micropollutants [13]. Consequently, the synthesis, modification, and application of carbon-based adsorbents such as activated carbon (AC) are likely to expand, as their low cost, porous architecture, and versatility align well with these treatment needs. However, most studies on the production and application of ACs do not assess their full environmental impact through ecotoxicity testing. If discharged improperly, these materials, or their leachates, may enter aquatic systems, potentially harming resident species and, ultimately, human health. In addition, to evaluate the carbon-based source on the impacts of benthic organisms, Lillicrap et al. [15] compared biogenic (1050 m^2^/g, from coconut shell) and petrogenic (900 m^2^/g from bituminous coal) AC using a tiered test battery approach. They found that *Chironomus riparius* was sensitive (reduction in development rate and time to first emergence) to low levels of biogenic AC (5% (*w*/*w*) dry weight). Similarly, higher doses of AC exposure resulted in a reduction in the survival of *Testudinella battagliai*, with biogenic materials inducing stronger negative responses compared to petrogenic AC. Biogenic AC and biochar are typically enriched with oxygen-containing functional groups (e.g., carboxyl, hydroxyl) capable of interacting chemically with aquatic organisms and influence toxicity (e.g., [25]). In addition, these materials may retain residual impurities that could leach into the environment following application and potentially affect water quality and organisms’ health (e.g., [12]).

**Figure 5:**
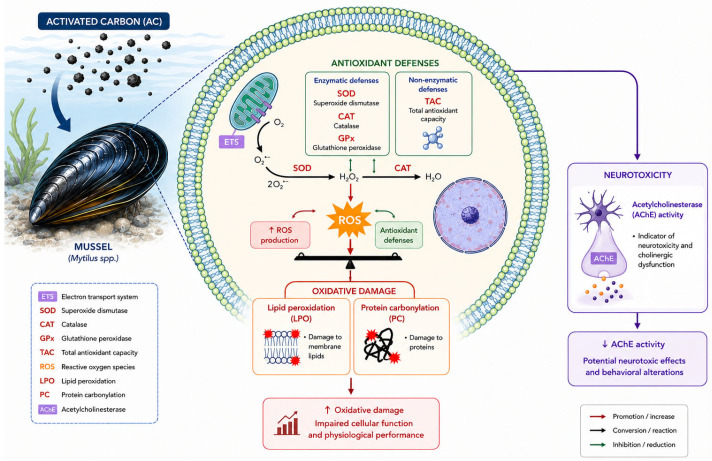
Schematic representation of the biochemical pathways and biomarkers assessed in *M. galloprovincialis* exposed to activated carbon derived from biomass (SBG-AC). The diagram summarizes responses related to energy metabolism, antioxidant defenses, oxidative damage, and neurotoxicity. Arrow colors were used to distinguish the type of biological reactions shown: red arrows indicate promotion or increase, black arrows indicate conversion or reaction pathways, and green arrows indicate inhibition or reduction.

Despite existing studies on the implications of AC use as a soil amendment, the impact of AC via alternative pathways (e.g., leaching from WWTPs) on invertebrates in marine/estuarine systems remains poorly explored. In addition, existing studies of these aquatic environments have reported inconsistent results, particularly with respect to sub-lethal physiological and biochemical responses (e.g., [16,20]). To improve the knowledge gap on the environmental impacts of AC through waterborne exposure (alternative pathways) in aquatic systems, the present study investigated how powdered SBG-AC (particle size ≤ 180 µm, doses of 5 to 50 mg/L), considered as adequate to be applied in the context of wastewater treatment, for removal of contaminants through adsorption tests (e.g., [9,10]), alters the biochemical status of widely used bioindicator species *M. galloprovincialis*, after chronic exposure. A scheme including the biomarkers assessed in this work is provided in Figure 5. The size range of the materials applied is within the particle-retention capacity reported for bivalves [38], suggesting that SBG-AC particles can be retained and potentially ingested by *M. galloprovincialis*.

Among the sub-lethal responses, the state of energy metabolism within organisms is an important indicator of growth and development under environmental stressors, helping to predict ecological consequences [36,39]. In the present study, the increase in metabolic activity (ETS activity) together with the expenditure of energy reserves (GLY) might indicate that the filtering capacity of the mussels was increased, especially at higher doses (50 mg/L) of SBG-AC. This material can interact with mussels from both direct and indirect mechanisms of action, as follows: due to its low size (powdered), AC particles can pass through filtration, retention on gills, ingestion and/or rejection. In addition, indirect effects may include reduced food availability or assimilation due to adsorption of organic matter/food components by SBG-AC and consequent energetic imbalance. In fact, in the present work, organisms seemed to allocate internal energy storage, using GLY, to overcome the reduced food assimilation. The use of internal energy reserves was already observed in studies with invertebrate’ organisms facing other stressors due to a mismatch between organismal energy demand and energy supply from the environment (e.g., [39,40]). Bivalves are particle feeders that can ingest particles, in general, up to 600–900 μm, although particle size, shape, and surface properties can influence whether particles are ingested or rejected as pseudofeces, i.e., particles expelled before entering the digestive tract and therefore not assimilated as food [38].

As reported by Rämö et al. [16], the co-ingestion of AC and sequestration of ingested substances may reduce the uptake of food assimilation by some species of benthic invertebrates as the bristle worm *Marenzelleria* spp., resulting in starvation. However, the literature on carbon-based materials and aquatic invertebrates’ filtering capacity remains limited and inconsistent. As an example, Woermann et al. [41] showed no impacts on the filtering capacity of bivalves (*Corbicula* sp.) exposed for 5 and 10 weeks to loaded (with micropollutants) powdered AC (PAC, collected from a WWTP) and to unloaded PAC, both at doses of 1, 10, and 100 mg/L. This was true even though the authors suggested that bivalves remove PAC by avoiding ingestion and producing pseudofeces.

Besides reflecting the filter-feeding capacity, the enhanced metabolic capacity (ETS) may also be associated with other physiological processes, such as antioxidant defenses or biotransformation mechanisms, both of which rely on protein synthesis, including enzymes and heat shock proteins (that prevent protein aggregation and re-establish functionality of denatured proteins) [39]. The enzymatic and non-enzymatic scavengers are thus essential in mitigating oxidative stress by neutralizing ROS and preventing cellular damage. In the present study, mussels exposed to lower SBG-AC doses showed similar or slightly higher activities of CAT, SOD, and TAC capacity, although these changes were not statistically significant (except for SOD at SBG-AC 25 mg/L). The absence of significant changes in these biomarkers may indicate that redox homeostasis was maintained under these exposure conditions, or that these enzymes were less responsive at the tested exposure time. Antioxidant enzymes are not always modulated simultaneously, as their activity depends on the type and amount of ROS produced, exposure duration, and cellular energy status. As an example, GPx activity depends on the availability of reduced glutathione, while CAT activity mainly contributes to hydrogen decomposition when this molecule accumulates at higher concentrations [29]. The significantly higher ETS activity at lower SBG-AC doses may indicate increased metabolic activity and energy demand, providing the necessary fuel to sustain antioxidant defenses and, at the same time, contributing to increased ROS production (e.g., superoxide anion and hydrogen peroxide), formed in the mitochondria [29,42]. At higher SBG doses (50 mg/L), the defense capacity in mussels seemed to be compromised, as the first line of defense against oxidative stress (SOD) exhibited a hormetic pattern, being inhibited at the highest concentration, and failing the conversion of superoxide anion into a stable molecule. In addition, the significant GPx activity decrease at higher SBG doses (25 mg/L) may indicate a possible limitation in reduced glutathione (GSH) availability, which is essential for its catalytic function [29]. As this enzyme acts in the first line of defense against moderate oxidative stress, under elevated hydrogen peroxide conditions or other peroxides (e.g., resulting from cellular damage) it may become inactivated [43]. However, this response was not clearly dose dependent, since values at 50 mg/L were similar to the lower SBG-AC dose (5 mg/L) and the control treatments. This indicates that GPx was less consistently affected than SOD. These non-parallel responses among antioxidant enzymes are common in aquatic organisms, as SOD, CAT, and GPx differ in substrate specificity, subcellular localization, regulatory mechanisms, and exposure level, resulting in distinct patterns of activation or inhibition under oxidative stress [44,45]. Lee et al. [46] observed similar inhibition of SOD activity in *D. magna*, under varying doses of iron (Fe)-doped biochar (0.12–1000 mg/L). Although this material is not directly comparable to the potassium-activated SBG-AC used here, as it was modified through iron incorporation rather than chemical activation, the findings suggest that exposure to modified carbonaceous materials can alter antioxidant defenses in aquatic organisms despite differences in composition and production methods. In fact, the SOD inhibition was related to higher levels of ROS/RNS, especially at lower doses of Fe-doped biochar, leading to oxidative damage to macromolecules. Moreover, the presence of iron, a redox-active metal that can undergo redox cycling reactions, contributes to additional reactive free radical production. In the present work, despite the inhibition of SOD and GPx at higher SBG-AC doses (50 mg/L), the other antioxidant enzyme (CAT) remained active, contributing to the decomposition of ROS and to the overall increase in TAC. Although the Fe-related redox cycling mechanism proposed is not expected to occur in the present study, the inhibition of SOD and GPx can be related with the exposure to SBG-AC, potentially involving particle-related effects, and other leachable constituents.

Despite all antioxidant efforts, the cellular defenses were overwhelmed, failing to neutralize the ROS surge induced by the higher SBG-AC doses. This response is supported by significantly higher LPO and PC levels, especially at the higher SBG-AC dose (50 mg/L), since these biomarkers are commonly associated with oxidative damage to lipids and proteins [29]. Nevertheless, the previously referred study by Woermann et al. [41] showed no changes induced by PAC (1–100 mg/L) on the antioxidant system (glutathione S-transferases, CAT, and SOD activities), and no cellular damage (LPO) was observed in bivalves *Corbicula* sp. The authors attributed this lack of toxicity either to absence of harmful effects under the tested conditions, or to avoidance of PAC via excretion as pseudofeces. Despite the dosage range overlap with that of the present study (SBG-AC: 5–50 mg/L), possibly differences in the type of AC and physical-chemical properties such as particle size and surface chemistry may have contributed to the observed toxicity, explaining the distinct outcomes.

Additionally, if ingested by these filter-feeding organisms, the presence of oxygen radicals (persistent free radicals, PFRs) on the surface of the carbon-based materials (formed during the materials’ production) may amplify ROS generation, potentially leading to oxidative damage [47,48]. In other species, however, these PFRs were reported to induce oxidative stress by the generation of ROS. Zhang et al. [49] demonstrated that biochar produced from ground pine needles significantly promoted the generation of cellular ROS in the algae *S. obliquus*, with the increase in dose (50–800 mg/L). In addition, the authors found that SOD activity was affected by biochar dose (significantly increased above 200 mg/L) and by temperature of pyrolysis (highest at 500 °C). In contrast, within water treatment contexts, these same PFRs can participate in the activation of oxidants (such as hydrogen peroxide, persulfate), leading to the generation of different free radicals for contaminant removal [50,51].

Besides oxidative stress, in the present study, no significant neurotoxicity was observed in mussels exposed to SBG-AC. The absence of marked response may suggest either limited bioavailability of potential neurotoxic compounds that could leach from SBG-AC matrix or their efficient detoxification by these organisms. This is true even though Lieke et al. [25] reported neurotoxicity, through impaired defecation and recognition, and response to a chemical attractant on the nematode *Caenorhabditis elegans* after exposure to biochar produced from rice straw (250–2000 mg/L). The authors related the toxicity to the presence of PFRs, which can destabilize cellular membranes and ultimately lead to cell death. In a related study, however, Dlamini and Otomo [52] reported neurotoxicity in *Eisenia fetida* (through alteration of acetylcholinesterase activity) following exposure to pine tree biochar used for soil amendment, with significant alterations at doses above 10% (*w*/*w*). The differences between these findings and the present study could be explained by species-specific susceptibility, reflecting distinct physiology and detoxification capacities towards neurotoxic compounds present in carbon-based materials. Under elevated doses of these materials (2000 mg/L, as in [25]), the contributions with potential neurotoxic compounds, including free radicals and ROS, are expected to increase and impair neuronal functions. In contrast, in the present study, the doses of SBG-AC and the overall contribution of ROS may not be enough to exert neurotoxicity.

Collectively, the results presented here show that, despite the proven efficacy of SBG-AC in water treatment (e.g., [9,10]), environmental leachate or discharge containing this material may adversely affect aquatic invertebrates, with responses depending on the dose. In addition, other studies reported that, beyond dose, particle size, and manufacturing conditions (precursor and temperature) can modulate the responses of organisms. Nevertheless, because particle ingestion or tissue accumulation was not directly determined in this work, the biological responses observed should be interpreted with caution, as they may result from exposure to SBG-AC particles and/or leachable constituents. In addition, parameters related to sex and reproductive stage were not assessed, but their influence on the biomarkers tested cannot be excluded. Although the use of whole soft tissue provided an integrated overview of the biochemical status of *M. galloprovincialis*, this approach does not allow the identification of organ-specific responses. In addition to these factors, future studies should also include intermediate time exposures, such as 7, 14, and 21 days, to better characterize the temporal progression of biological responses and understand the mechanism of action associated with carbon-based materials.

These considerations underscore the need for ecotoxicity assessments before material application. As previously referred to, SBG-AC is usually applied at doses ranging from 5 to 50 mg/L for pharmaceutical removal in aqueous systems; however, these doses may increase in complex matrices such as wastewater due to competitive adsorption effects [10]. Proper containment and application of SBG-AC at optimized doses are thus important to guarantee that the use of SBG-AC can be beneficial by removing pharmaceutical drugs while minimizing ecological risks.

## 4. Materials and Methods

### 4.1. Reagents and Chemicals

Potassium carbonate (K_2_CO_3_, 99.9%) was acquired from AnalaR Normapur. Hydrochloric acid (HCl, 37.0%) was obtained from Honeywell FLUKA. Potassium phosphate buffer was prepared using dipotassium hydrogen phosphate (K_2_HPO_4_, 99%, PanReac AppliChem, Barcelona, Spain) and potassium dihydrogen phosphate (KH_2_PO_4_, 98%, Thermo Fisher Scientific, Waltham, MA, USA). Ethylenediaminetetraacetic acid (EDTA, Scharlab, Sentmenat, Spain) was used at the manufacturer’s specified purity. Triton™ X-100 (Sigma-Aldrich, Burlington, MA, USA), a non-ionic surfactant consisting of a mixture of oligomeric compounds, was used as supplied. Dithiothreitol (DTT, 99.5%, PanReac AppliChem) was used as the reducing agent. Tris-HCl buffer was prepared from Tris base (>99.8%, Fisher Scientific), with pH adjusted using HCl. Polyvinylpyrrolidone (PVP, TCI Chemicals, Tokyo, Japan), a polymer for which no purity percentage is provided by the manufacturer, was used as received. Magnesium sulfate heptahydrate (MgSO_4_·7H_2_O, >99%, Thermo Fisher Scientific), trichloroacetic acid (TCA, 99%, CHEMSOLUTE, Renningen, Germany), and thiobarbituric acid (TBA, >98%, TCI Chemicals) were also used throughout the biochemical assays.

### 4.2. Activated Carbon Production

The activated carbon (AC) from spent brewery grain (SBG) (SBG-AC) materials used in this work was produced according to Sousa et al. [9]. Firstly, the precursor materials, SBG, were collected in the Brewery Faustino Microcervejeira, Lda (Aveiro, Portugal), and transported to the laboratory. The SBG was dried in an oven (J.P. Selecta, Barcelona, Spain) at 105 °C, and ground with a blade mill. Then, the dried material was impregnated with the activating agent potassium carbonate (K_2_CO_3_) in a 1:2 activating agent/SBG ratio (*w*/*w*). The impregnated material was stirred for 1 h in an ultrasonic bath, and later left to dry at room temperature. After, the dried impregnated SBG was carbonized under N_2_ atmosphere (~100 mLn/min) in a microwave oven (CEM Phoenix™ AirWave, CEM Corporation, Matthews, NC, USA), heated at a rate of 15 °C/min until 800 °C, with a residence time of 20 min. The resulting materials were washed with ~1.2 M hydrochloric acid (HCl) followed by distilled water until the aqueous leachate reached neutral pH. After, the washed carbonized materials were separated from the washing leachates by filtration (filter paper, 7–12 µm, from Macherey-Nagel (Düren, Germany), reference 222009) and were dried overnight at 105 °C. When dried, the materials were crushed and sieved to obtain a fine powder (particle size ≤ 180 µm). As previously reported, this fraction was selected based on previous wastewater treatment studies using the same material and comparable particle size. No further particle-size distribution analysis was performed within the fraction used; therefore, the relative contribution of smaller particle-size classes is not available. However, particles smaller than 7–12 µm are not expected to be present in significant amounts, as the SBG-AC was recovered using filter paper with this pore-size range, as described above.

The powder (SBG-AC) was stored in a desiccator at room temperature until use. The materials produced had previously been characterized for their textural and chemical properties, including scanning electron microscopy (SEM), X-ray photoelectron spectroscopy (XPS), and point of zero charge (PZC), as reported in previous studies [8,9,10].

### 4.3. Experimental Conditions

*Mytilus galloprovincialis* (Lamarck, 1819) specimens were selected as the bioindicator species for this study. The organisms were collected from the Mira channel (40°36′55″ N 8°44′25″ W) on the coastal lagoon of Ria de Aveiro (Aveiro, Portugal) in November 2024, outside the main reproductive period for this species in the study area. After collection, the organisms were transported to the laboratory where they were cleaned and maintained for 10 days under constant aeration and conditions similar to the sampling site (temperature 17.0 ± 1.0 °C; salinity 30 ± 1; pH 8.0 ± 0.1; natural photoperiod). Artificial seawater was prepared using a commercial salt (Salt Red Sea) and deionized water. During the acclimation period, artificial water was renewed every 2–3 days, according to water quality, namely visible accumulation of feces, pseudofeces, and organic matter. The mussels were fed with a mixture in equal parts of three algae, as follows: *Isochrysis galbana*, *Tetraselmis* sp., *Phaeodactylum tricornutum* (only after three days of acclimation, then three times per week with 150,000 cells/animal/day).

After the acclimation period, five mussels were placed in 3 L aquaria (three aquarium per treatment), filled with artificial seawater (temperature 17.0 ± 1.0 °C; salinity 30 ± 1; pH 8.0 ± 0.1). Mussels of similar size were selected for the experiment, with shell dimensions of 60 ± 4 mm (length) and 36 ± 3 mm (width), to minimize variability associated with different growth stages.

The mussels were randomly distributed by four treatments with different doses of SBG-AC for a period of 28 days, as follows: control (CTL, clean artificial seawater with no SBG-AC), 5 mg/L, 25 mg/L, and 50 mg/L of SBG-AC. The suspension was obtained by adding a determined mass of SBG-AC (powder, ≤ 180 µm) to the artificial water in the aquaria. During the exposure, partial settling of SBG-AC was observed, especially in SBG-AC 25 and 50 mg/L, despite the constant aeration that contributed to maintaining the medium and material in suspension. The doses of SBG-AC chosen for this study were selected based on previous studies in which SBG-AC was used as an adsorbent for contaminants, particularly pharmaceutical drugs (e.g., [9,10]). Mussels were fed with the same algae composition of the acclimation period (150,000 cells/animal/day, three times per week), and the seawater was renewed every week. The conditions of temperature, salinity, pH, photoperiod, and the SBG-AC doses were re-established after each water renewal. At the end of exposure, the organisms were removed from the aquaria, frozen, and stored at −80 °C until further analysis.

No mortality was observed in the treatments tested during the experiment (28 days).

### 4.4. Biomarker Analyses

Biomarker analyses were determined in the whole soft tissues of *M. galloprovincialis* (three organisms per aquarium, nine per treatment), using protocols already described in the literature [32,53,54]. General stress response parameters related with energy metabolism and oxidative stress were evaluated in mussel tissue samples as follows: indicators of energy metabolism (electron transport system activity, ETS; glycogen content, GLY; total protein content, PROT); antioxidant capacity (total antioxidant capacity, TAC; activities of the enzymes: superoxide dismutase, SOD; catalase, CAT; glutathione peroxidase, GPx); cellular damage (lipid peroxidation levels, LPO; protein carbonylation levels, PC); and neurotoxicity (acetylcholinesterase activity, AChE). Whole frozen soft tissue from each mussel was manually homogenized using a mortar and a pestle under liquid nitrogen and divided into 0.5 g fresh weight (FW) aliquots for the biochemical analyses. Each aliquot was submitted to extraction, with specific extraction buffers, in the ratio of 1:2 (*w*/*v*). Specifically, potassium phosphate buffer (50 mM potassium phosphate, pH 7, ethylenediaminetetraacetic acid 1 mM, 1% (*v*/*v*) TritonX-100, and 1 mM dithiothreitol) was used for the extraction of PROT, GLY, TAC, SOD, CAT, GPx, PC, and AChE. For the determination of ETS, a Tris-HCl buffer was applied (0.1 mM Tris-HCl, pH 8.5, containing 15% (*w*/*v*) polyvinylpyrrolidone, 153 mM magnesium sulfate, 0.2% (*v*/*v*) Triton X-100). Finally, trichloroacetic acid (TCA) buffer (20% (*v*/*v*) TCA with 0.5% (*w*/*v*) thiobarbituric acid was used for LPO levels determination. For extraction, tissue samples were homogenized with corresponding buffers for 90 s using a TissueLyser II (Qiagen, Hilden, Germany), followed by centrifugation (centrifuge VWR Mega Star 600R, VWR International, Radnor, PA, USA) (4 °C, 10,000× *g* (or 3000× *g* for ETS), 20 min). The supernatant was collected and stored at −80 °C until analysis, following protocols described elsewhere [32,53,54]. Detailed protocols for the biochemical analyses performed are described in the Supporting Information (SI—Section S1). The samples were analyzed in duplicate in a microplate reader (Synergy™HT, Biotek Instruments, Inc., Winooski, VT, USA).

### 4.5. Data Analyses

The biomarker responses obtained were subjected to hypothesis testing through permutational analysis of variance, performed with the PERMANOVA routine in PRIMER v7.0.10 software (PRIMER-e, Auckland, New Zealand) [55]. The analysis tested the null hypothesis that no significant differences exist among SBG-AC treatments (CTL, 5, 25, and 50 mg/L of SBG-AC) after 28 days of exposure. When the main PERMANOVA test was significant (*p* < 0.05), pairwise comparisons were performed as post hoc tests to identify differences among treatments, i.e., to identify which SBG-AC treatments differed from the control and/or from each other. No additional correction for multiple comparisons was applied. For each biomarker, homogeneity of dispersion among SBG-AC treatments was assessed using PERMDISP routine also on the PRIMER v7.0.10. PERMDISP results were used to verify whether significant PERMANOVA results could be attributed to differences among treatments rather than to unequal dispersion within groups. The resulting PERMANOVA and PERMDISP *p*-values are reported in the Supporting Information (Section S2), in Table S1. Significant differences among SBG-AC treatments, when detected, are identified in the Figures with different lowercase letters.

## 5. Conclusions

The results of the present study showed that SBG-AC, obtained by microwave pyrolysis and chemical activation of a lignocellulosic biomass, altered the energy-related metabolism and the antioxidant capacity of mussels *M. galloprovincialis*, used here as a bioindicator species, after a chronic exposure. Significant impacts were mostly observed at the highest dose tested (SBG-AC 50 mg/L). These findings highlight the importance of evaluating the potential biological effects of activated carbons before environmental application. When properly contained during operation and appropriately disposed of, their potential environmental impacts can be minimized. By characterizing dose-dependent responses and delineating exposure levels associated with minimal effect, this study advances understanding of the ecotoxicity of carbon materials and informs the safer, more sustainable deployment of waste-derived activated carbons in remediation strategies. 

## Data Availability

The datasets generated during and/or analyzed during the current study are available from the corresponding author on reasonable request.

## Supplementary Materials

The following supporting information can be downloaded at: https://www.mdpi.com/article/10.3390/ijms27146358/s1. References [56,57,58,59,60,61,62,63,64,65,66,67,68] are cited in the Supplementary Material.

## Abbreviations

The following abbreviations are used in this manuscript:

AC	Activated Carbon
AChE	Acetylcholinesterase
CAT	Catalase
ETS	Electron Transport System
FW	Fresh Weight
GLY	Glycogen
GPx	Glutathione Peroxidase
LPO	Lipid peroxidation
PAC	Powdered Activated Carbon
PC	Protein carbonylation
PFRs	Persistent Free Radicals
PROT	Total protein
ROS	Reactive Oxygen Species
SBG	Spent Brewery Grains
SBG-AC	Spent Brewery Grains—Activated Carbon composite
SOD	Superoxide dismutase
TAC	Total antioxidant capacity
WWTP	WasteWater Treatment Plant
