# Supplementary material for: Ecotoxicological Effects of a Biomass-Derived Carbon Adsorbent on the Mussel Mytilus galloprovincialis
Source: Int J Mol Sci. 2026 Jul 17;27(14):6358. doi: 10.3390/ijms27146358 (PMC13409804; doi:10.3390/ijms27146358)
Supplement: Supplementary file 1 [file ijms-27-06358-s001.zip › ijms-4359995-supplementary.pdf]

SUPPORTING INFORMATION

Ecotoxicological effects of a biomass-derived carbon adsorbent on the mussel *Mytilus galloprovincialis*

Almeida, Â.[1,*], Canha, T.[2], Cunha, M.[3], Calisto, V.[1], Freitas, R.[3]

1 Department of Chemistry and CESAM, University of Aveiro, 3810-193, Aveiro, Portugal

2 Department of Physics, University of Aveiro, 3810-193, Aveiro, Portugal

3 Department of Biology and CESAM, University of Aveiro, 3810-193, Aveiro, Portugal

** E-mail: aaalmeida@ua.pt*

Corresponding author: Ângela Almeida, Chemistry Department & CESAM, University of Aveiro, Campus Universitário de Santiago, 3810-193 Aveiro, Portugal, aaalmeida@ua.pt

1. **Biomarker analyses**

1.1. Indicators of energy metabolism

ETS (electron transport system) activity was determined based on the method of Owens & King [56], with modifications by De Coen & Janssen [57]. In this parameter, NADPH (nicotinamide adenine dinucleotide phosphate) is used as the electron donor, leading to the reduction of the dye INT (p-iodonitrotetrazolium, 2-(4-iodophenlyl)-3-(4-nitrophenyl)-5-phenyl-2H-tetrazolium chloride) and formation of a colored formazan product. Absorbance was measured at 490 nm for 10 min at 25 s intervals. The results were expressed as mol of formazan formed per minute per gram of fresh weight (nmol/min/g FW).

GLY (glycogen) content was quantified using the total sugar assay described by DuBois et al. [58]. This method is based on the reaction of total sugars in samples and standards with phenol in an acidic medium, forming a colored complex, whose intensity is proportional to sugar concentration. For determination, a calibration curve was prepared using glucose standards (0.10 – 5 mg/mL). Absorbance was measured at 490 nm after allowing the reaction mixture to develop for 30 min at room temperature. The results were expressed as mg per gram of fresh weight (mg/g FW).

PROT (total protein) content was quantified using the biuret complex method, described by Robinson & Hodgen [59]. The method relies on the formation of a colored complex in alkaline medium containing sodium-potassium tartrate, in which copper (II) ions reacts with peptide bonds of aminoacids present in samples and standards. A calibration curve was prepared using bovine serum albumin (BSA) standards (5 – 40 mg/mL). Reaction mixtures were incubated for 10 min at 30 ºC, in the dark. Absorbance values were recorded at 540 nm. Results were expressed as mg of protein per gram fresh weight (mg/g FW).

1.2. Antioxidant capacity

TAC (total antioxidant capacity) levels were evaluated using the FRAP (Ferric Reducing Antioxidant Power) assay described by Benzie & Strain [60], with modifications made by Hagger et al. [61]. This method is based on the reduction of ferric (Fe^{3+}) to ferrous (Fe^{2+}) ions in an acidic medium by antioxidants present in sample, forming a colored ferrozine complex measured by absorbance at 593 nm, following a 10 min incubation reaction. A calibration curve was prepared with standards (50 – 1000 µM) of iron (II) sulphate heptahydrate ($FeSO_4 \cdot 7H_2O$). Results were expressed as µmol Fe^{2+} formed per gram fresh weight (µmol/g FW).

SOD (superoxide dismutase) activity was determined according to Marklund & Marklund [62]. This method is based on the ability of SOD in samples to inhibit the rate of pyrogallol auto-oxidation, which occurs under alkaline conditions and generates the superoxide radical. Absorbance was read at 420 nm and expressed as the amount of enzyme required to inhibit 50 % of pyrogallol auto-oxidation per gram of fresh weight (U/g FW).

CAT (catalase) activity was determined according to the method of Aebi [63]. In this assay, CAT in the samples catalyzes the decomposition of hydrogen peroxide to water and molecular oxygen. The absorbance of hydrogen peroxide decomposition was measured at 240 nm over 2 min with 15-s intervals. The activity was expressed as U (amount of enzyme that caused the decomposition of 1 µmol of hydrogen peroxide per min) per gram of fresh weight (U/g FW).

GPx (glutathione peroxidase) activity was measured according to Paglia & Valentine [64]. The decrease in absorbance of the reaction mixture was monitored at 340 nm for 5 min, with readings every 15 s. Enzymatic activity was expressed as U/g FW, where U corresponds to the oxidation of 1 µmol NADPH per minute per gram of fresh weight (FW).

1.3. Indicators of cellular damage

LPO (lipid peroxidation) levels were quantified as thiobarbituric acid-reactive substances (TBARS) according to Buege & Aust [65]. Samples were incubated with 0.5 % (*w/v*) 2-thiobarbituric acid and 20 % trichloroacetic acid at 96 ºC for 25 min. Absorbance was read at 532 nm. The concentration of malondialdehyde (MDA), a terminal product of LPO, was calculated, and the results were expressed as nmol MDA per gram of fresh weight (nmol MDA/g FW).

PC (protein carbonylation) levels were determined following Mesquita et al. [66], using 2,4-dinitrophenylhydrazine (DNPH) that reacts with protein carbonyl groups to form hydrazone derivatives. After derivatization with DNPH, the reaction was neutralized with sodium hydroxide, and absorbance was measured at 450 nm. Results were expressed as nmol of carbonyl groups formed per gram of fresh weight (nmol/g FW).

1.4. Indicator of neurotoxicity

AChE (acetylcholinesterase) activity was determined following the method of Ellman et al. [67], with adaptations from Mennillo et al. [68]. Acetylthiocholine iodide (ATChI, 5 mM) and 5,5'-dithiobis(2-nitrobenzoic acid) (DTNB) were used as substrate and chromogenic reagent, respectively. The reaction was monitored at 412 nm for 5 min with readings taken at 1 min intervals. Enzyme activity was expressed as nmol of 5-thio-2-nitrobenzoate product (colored) formed per minute per gram of fresh weight (nmol/min/g FW).

2. **Data Analysis**

Table S1. Pairwise PERMANOVA *p*-values for biomarker responses measured in *Mytilus galloprovincialis* after 28 days of exposure to SBG-AC treatments: control, 5, 25 and 50 mg/L. Biomarkers included ETS, electron transport system; GLY, glycogen content; PROT, total protein content; TAC, total antioxidant capacity; SOD, superoxide dismutase; CAT, catalase; GPx, glutathione peroxidase; LPO, lipid peroxidation; PCO, protein carbonylation; and AChE, acetylcholinesterase). Pairwise comparisons were performed when the main PERMANOVA test was significant ($p < 0.05$). Significant *p*-values for the pairwise conditions are indicated in bold. No significant differences were observed among treatments for PROT, CAT, and AChE; as the main PERMANOVA results was not significant. PERMDISP was used to assess the homogeneity of dispersion among treatments. Non-significant PERMDISP *p*-values indicate no evidence of heterogeneous dispersion.

**Pairwise condition**	**ETS**	**GLY**	**PROT**	**TAC**	**SOD**	**CAT**	**GPx**	**LPO**	**PC**	**AChE**
**CTL *vs* 5**	**0.0241**	0.0717	0.5523	0.2712	0.9445	0.2698	0.6025	0.1091	0.1718	0.1242
**CTL *vs* 25**	**0.0081**	0.1112	0.2060	0.0578	0.0692	0.1038	**0.0334**	**0.0458**	0.1023	0.6282
**CTL vs 50**	**0.0093**	**0.0220**	0.2799	**0.0293**	**0.0275**	0.0998	0.0521	**0.0244**	**0.0351**	0.1025
**5 *vs* 25**	0.5220	0.8388	0.8909	0.3096	**0.0480**	0.2193	0.1667	0.2466	0.7809	0.2928
**5 *vs* 50**	0.0655	0.5534	0.6176	0.0629	**0.0157**	0.2148	0.2821	0.0723	0.1159	0.3683
**25 *vs* 50**	0.0704	0.9024	0.6335	0.1528	**0.0063**	0.8979	0.3144	0.3170	0.1059	0.1659
**PERMDISP**	0.0533	0.2340	0.1513	0.5000	0.5869	0.4091	0.4963	0.5116	0.3167	0.2294
